# The sociolinguistic foundations of language modeling

**DOI:** 10.3389/frai.2024.1472411

**Published:** 2025-01-13

**Authors:** Jack Grieve, Sara Bartl, Matteo Fuoli, Jason Grafmiller, Weihang Huang, Alejandro Jawerbaum, Akira Murakami, Marcus Perlman, Dana Roemling, Bodo Winter

**Affiliations:** Department of Linguistics and Communication, University of Birmingham, Birmingham, United Kingdom

**Keywords:** AI ethics, artificial intelligence, computational sociolinguistics, corpus linguistics, large language models, natural language processing, varieties of language

## Abstract

In this article, we introduce a sociolinguistic perspective on language modeling. We claim that language models in general are inherently modeling *varieties of language*, and we consider how this insight can inform the development and deployment of language models. We begin by presenting a technical definition of the concept of a variety of language as developed in sociolinguistics. We then discuss how this perspective could help us better understand five basic challenges in language modeling: *social bias, domain adaptation, alignment, language change*, and *scale*. We argue that to maximize the performance and societal value of language models it is important to carefully compile training corpora that accurately represent the specific varieties of language being modeled, drawing on theories, methods, and descriptions from the field of sociolinguistics.

## 1 Introduction

The underlying task of language modeling is to predict the probability of word tokens, or other linguistic forms, in a text based on previously observed texts (Jurafsky and Martin, 2023). Language modeling is not new (Bengio et al., 2003), but when pursued through the analysis of extremely large corpora of natural language using transformer-based architectures (Vaswani et al., 2017; Devlin et al., 2018), it has proven to be a uniquely effective approach to natural language processing (NLP) (Radford et al., 2019). These systems, which have come to be known as Large Language Models (LLMs), are currently revolutionizing Artificial Intelligence (AI), with especially powerful LLMs such as GPT-4 (Achiam et al., 2023), LLaMa (Touvron et al., 2023), Mistral (Jiang et al., 2023) often being referred to as *base models* or *foundation models* (Bommasani et al., 2021) due to their high levels of fluency and their ability to help achieve state-of-the-art performance across a wide range of downstream tasks, most famously in chatbots like ChatGPT (Ray, 2023). Despite increasing concerns about the risks of LLMs (Bender et al., 2021), experts across many fields believe they will have a major impact on society, including in medicine (Thirunavukarasu et al., 2023; Huang Y. et al., 2024), education (Kasneci et al., 2023; Yigci et al., 2024), computer programming (Li et al., 2022; Wang et al., 2024), journalism (Pavlik, 2023; Li et al., 2024), economics (Horton, 2023; Guo and Yang, 2024), and technical writing (Lund et al., 2023; Cruz-Castro et al., 2024).

Given the growing societal importance of LLMs, language modeling has provoked critical discussion from a wide range of perspectives, not only AI and NLP (e.g., Bender et al., 2021; Bommasani et al., 2021; Jiao et al., 2024; Head et al., 2023), but in linguistics (e.g., Piantadosi, 2023; Dentella et al., 2023; Marcus et al., 2023), cognitive science (e.g., Hardy et al., 2023; Demszky et al., 2023; Michaelov et al., 2024), and ethics (e.g., Birhane et al., 2023; Cabrera et al., 2023; Li et al., 2023; Stefan et al., 2023; Haque and Li, 2024). There is, however, a very basic question about language models that has received remarkably little attention in the literature:

What is actually being modeled by language models?

Although the goal of language modeling is clear (i.e., token prediction), the type of language being modeled by language models is usually only defined in the most general terms, for example, “a broad swath of internet data” (Brown et al., 2020). Models are often trained on corpora based at least in part on the CommonCrawl dataset or alike (Radford et al., 2019; Raffel et al., 2020; Baack, 2024), but otherwise, in most cases, the nature of the language being modeled is not described at all (Bender et al., 2021). In large part, this is a natural consequence of the need for massive amounts of data to train base models, making the sources of these corpora of secondary concern. However, even when these models are adapted for more specific contexts (Gururangan et al., 2020), the type of language used for further training is generally only loosely defined. For example, ChatGPT was developed by adapting a GPT-3.5 base model for dialogue (OpenAI, 2022), but the form of dialogue actually being modeled by ChatGPT is something much less diverse and much more artificial than everyday English conversation, as anyone who interacts with ChatGPT knows.

Drawing on modern sociolinguistic theory, in this paper, we therefore provide an answer to the question what is being modeled by language models?

Language models are models of ***varieties of language***.

We argue that any language model is inherently modeling the variety of language represented by the corpus on which it is trained, even if that variety of language is unknown and even if that corpus is a poor representation of that variety of language. Our view is that this simple insight can inform, at a fundamental level, how language models are developed and deployed. Given rapid advances in language modeling in recent years and the increasing societal impact and risk associated with LLMs, we believe the sociolinguistic perspective we are proposing in this paper is especially important at this time—not only to improve the performance, evaluation, and applicability of LLMs, but to guide the creation of safe and ethical AI systems and to help us better understand their underlying nature.

In the rest of this paper, we expand on our claim that, in its basic form, a language model of any type represents a variety of language, and we consider the implications of this claim for the task of language modeling. We do this primarily by synthesizing recent research in NLP and sociolinguistics, especially research from the emerging field of *computational sociolinguistics*, which sits at their intersection (Nguyen et al., 2016; Eisenstein, 2017; Grieve et al., 2023). We first provide a technical definition of the sociolinguistic concept of a variety of language and argue that this concept inherently underpins the task of language modeling. We then introduce and discuss five general challenges in language modeling that we believe the sociolinguistic perspective introduced in this paper can help address. We refer to these challenges as *social bias, domain adaptation, alignment, language change*, and *scale*.

Our primary goal in this position paper is therefore to introduce a sociolinguistic perspective on language modeling and to argue for its relevance to our general understanding of language models, as well as their development and deployment in the real world. Our intent is not to provide simple or specific solutions to major challenges in language modeling. Rather, our intent is to offer a new and general theoretical perspective from which to better understand these challenges, arguing for greater engagement in the field of language modeling with the field of sociolinguistics. Our core argument is that, when pretraining or further pretraining language models, it is important to carefully consider the specific varieties of language being modeled and to compile corpora that accurately represent these varieties of language. Furthermore, we argue that corpus compilation should be firmly grounded in theories, methods, and findings of sociolinguistics, which has long focused on understanding the nature of language variation and change. Our hope is that the proposals made in this paper will inspire future empirical research in language modeling, ultimately leading to improvements in the performance of language models and the societal value of the NLP systems into which they are embedded.

## 2 Defining varieties of language

A *variety of language*, or more simply a *variety*, is a term commonly used across linguistics to refer to any type of language (Crystal and Davy, 1969; Hartmann and Stork, 1972; Matthews, 1997; McEnery et al., 2006; Jackson, 2007; Crystal, 2011). The term is especially common in fields that study language variation and change—like sociolinguistics, dialectology, typology, historical linguistics, discourse analysis, stylistics, and corpus linguistics—where it is generally used to identify the types of language targeted for description, comparison, or other forms of linguistic analysis.

One reason a variety of language is such a powerful concept is because it can be used to identify such a wide range of phenomena—from very broadly defined varieties like the entire English language to very narrowly defined varieties like the speeches of a single politician. This terminology also allows linguists to sidestep debates, which are often underlyingly political in nature, like whether a given variety qualifies as a dialect or a language (Meyerhoff, 2018). For example, regardless of whether Scots is considered to be a dialect of English or a distinct language, Scots can be considered to be a variety, as well as a sub-variety of some larger Anglic variety that also includes English (Aitken, 1985). Similarly, regardless of whether Chinese is considered to be a family composed of many languages or a language composed of many dialects, all forms of Chinese can be considered to be both varieties themselves and part of some larger Sinitic variety (Huang H. et al., 2024).

**Figure 1:**
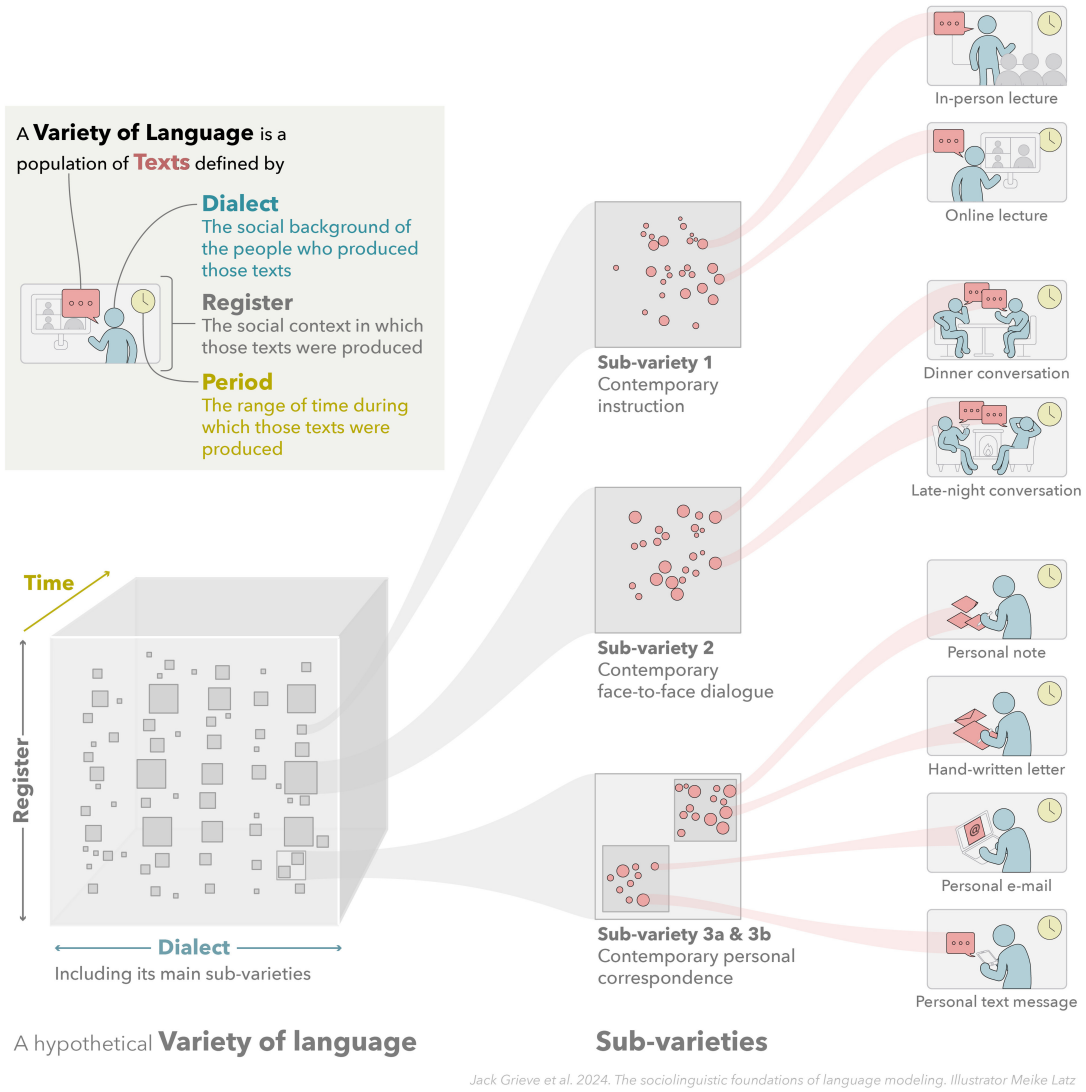
Varieties of language. This figure illustrates the concept of a variety of language, showing how the interaction between three distinct extra-linguistic factors—the social background of people who produce language (dialect), the social context in which language is produced (register), and the range of time over which language is produce (period)—can be used to specify a variety of language. It also illustrates how varieties of language are hierarchically organized, composed of smaller and smaller sub-varieties.

Although what are traditionally considered entire languages like English or Chinese can be referred to as varieties, the term is most commonly used in linguistics to refer to more narrowly defined sub-types of these larger languages (Crystal, 2011; Meyerhoff, 2018; Wardhaugh and Fuller, 2021). Such varieties are referred to by a wide range of technical and colloquial terms, including not only *dialects*, but *accents, sociolects, topolects, argots, jargons, registers, genres, styles, slangs, standards, periods*, and *eras*. We believe, however, that it is especially insightful to recognize three basic and distinct types of varieties—or, alternatively, three basic and distinct sources of linguistic variation—which we refer to as *dialect, register*, and *period/time* (see Figure 1).

**Dialects** are varieties defined by the social backgrounds of the people who produce language (Chambers and Trudgill, 1998; Meyerhoff, 2018; Wardhaugh and Fuller, 2021). Dialects are often associated with language that originates from speakers from particular nations, regions, classes, or ethnicities. Empirical research in sociolinguistics and dialectology has long shown that the language use of people from different social groups (Tagliamonte, 2006, 2011) and identities (Eckert, 2012, 2018; Ilbury, 2020) is characterized by systematic patterns of linguistic variation, especially variation in accent and vocabulary. For example, William Labov and his colleagues have analyzed variation in the pronunciation of American English in great detail (Bell et al., 2016; Gordon, 2017), from variation across class and other demographic variables in the pronunciation of /r/ post-vocalically in New York City (Labov, 1986, 1973) to mapping regional variation in the pronunciation of the entire English vowel system across North America (Labov et al., 2006). Lexical variation has also notably been the focus of considerable recent research in computational linguistics, primarily based on large corpora of social media (Donoso and Sánchez, 2017; Grieve et al., 2019; Huang et al., 2016; Bamman et al., 2014). For example, Blodgett et al. (2016) introduced a method for identifying lexical variation characteristic of African American English on Twitter, while also showing how NLP tools consistently underperform when applied to this dialect.

Alternatively, **registers** are varieties defined by the communicative contexts in which people, potentially from any social background, produce language (Biber and Conrad, 2019; Meyerhoff, 2018; Wardhaugh and Fuller, 2021). Registers are often associated with language produced in specific modalities, media, settings, and topics. It is important to stress that registers and dialects are independent: dialects are defined by the social backgrounds of language users, whereas registers are defined by the social contexts in which language users, regardless of their social backgrounds, communicate. Like dialect variation, there has been a long tradition of empirical research on register variation, predominantly in corpus linguistics (Biber, 1991; Sardinha and Pinto, 2014; Biber and Conrad, 2005) and discourse analysis (Martin, 2001; Matthiessen, 2015; Halliday, 1989), which has shown that language use across contexts is characterized by systematic patterns of linguistic variation, especially grammatical variation (Biber and Conrad, 2019). For example, Douglas Biber and his colleagues have studied register variation in English (Biber, 1991) and other languages (Biber, 1995) in great detail through the multivariate analysis of grammatical patterns across a range of corpora. Also, like dialect variation, recent research has focused on the analysis of large corpora of online language, especially social media data (Biber and Egbert, 2018; Clarke and Grieve, 2017; Liimatta, 2019; Pavalanathan and Eisenstein, 2015; Berber Sardinha, 2018). For example, Clarke (2022) described register variation in a corpus of English Twitter data through a multivariate analysis of grammatical features, identifying four general dimensions of stylistic variation.

Finally, **periods** are varieties defined by the time span over which language is produced (Nevalainen and Raumolin-Brunberg, 2016). Like dialects and registers, linguistic variation over **time** is also systematic. The study of *language change* has been one of the oldest endeavors in linguistics (Bybee, 2015; Campbell, 2013; Joseph et al., 2003; Lehmann, 2013). This research, which is also referred to as *historical linguistics*, has focused both on determining how mutually unintelligible varieties are historically related to each other and on describing how individual varieties, like English, have changed over time. Notably, recent research in computational sociolinguistics has studied how language changes over very short time spans based on large corpora of timestamped social media data, especially to analyze lexical innovation (Eisenstein et al., 2014; Grieve et al., 2017; Kershaw et al., 2016; Stewart and Eisenstein, 2018) For example, Grieve et al. (2018) showed how new words in American English tended to originate from five hubs of lexical innovation through a spatial analysis of a multi-billion-word corpus geolocated of Twitter data from across the US.

Taken together, these three extra-linguistic sources of linguistic variation allow for varieties of language to be defined with great flexibility and precision. This is illustrated in Figure 1, which shows how language use can be mapped across these three dimensions of linguistic variation, and how a variety of language can defined by taking into consideration the social background of people who produce language (dialect), the social context in which language is produced (register), and the range of time over which language is produced (period).

As Figure 1 illustrates, the relationships between varieties can be highly complex. Varieties can be defined at any scale and are generally hierarchically structured, being divisible into smaller and smaller sub-varieties. For example, English is a variety, but it also contains many smaller sub-varieties. These include many dialects, including national varieties of English, like British and American English, which are themselves composed of many smaller regional dialects like West Country English in the UK or African American English in the US (Chambers and Trudgill, 1998). At the most narrowly defined level, the language of an individual can be considered a distinct dialect (i.e., an idiolect). Similarly, English also includes many registers, including spoken and written English, which are themselves composed of many smaller registers, like conversations, telephone conversations, and personal telephone conversations (Biber and Conrad, 2019).

Along with exhibiting hierarchical structure, varieties can also be defined based on the overlap of larger varieties, as is also illustrated in Figure 1. For example, it is common to define a variety of interest by specifying a dialect, register, and period, like *Contemporary Conversational Canadian French* or *Scottish Novels from the Twentieth Century Written by Women*. In other words, we can think of a variety as being defined by the specification of one or more extra-linguistic factors related to the circumstances in which language is produced. In addition, the boundaries between varieties are not necessarily sharp or fixed. For example, one regional dialect or literary register might transition gradually into the next and this may change over time. For this reasons, sociolinguists often treat dialect, register, and time as dimensions of linguistic variation as opposed to hard categories.

Although we have defined a variety of language as a type of language, it is important to specify what exactly a variety of language consists of. In other words, when linguists study a variety of language, what are they actually studying? For many linguists, a variety of language is essentially a population of texts (or utterances), as circumscribed by one or more extra-linguistic factors, in particular, by a specific dialect, register, and period (see Croft, 2000). Notably, in this case, a **text** is broadly defined as the language (e.g., utterances, discourse) produced during any communicative event, including language produced in any modality (e.g., speech, writing, signing) (Halliday and Hasan, 1976). For example, not only can an email or an essay be considered a text, but so can a conversation or a speech. If we adopt what is known as an *externalist* approach to linguistics (Scholz et al., 2024; Sampson, 2002), where language in general is defined as the population of all texts (or utterances) that have ever been produced, a variety of language can be defined as a sub-population of those texts that meets some external definition—i.e., the totality of language produced by people from a particular social background (dialect), in a particular social context (register), and over a particular period of time (period).

For example, *Contemporary Spoken French Canadian Conversation* can be considered a variety of language, as it is a population of texts (i.e., conversations) produced by individuals from a specific social background (i.e., people who live in Canada), in a specific social context (i.e., spoken interactions), during a specific period (i.e., now). Similarly, a more narrowly defined type of language like *Scottish Novels from the Twentieth Century Written by Women* can also be considered a variety of language, as it is a population of texts (i.e., books) produced by individuals from a specific social background (i.e., female authors from Scotland), in a specific social context (i.e., long-form fictional narratives), during a specific time span (i.e., 1900-1999).

**Figure 2:**
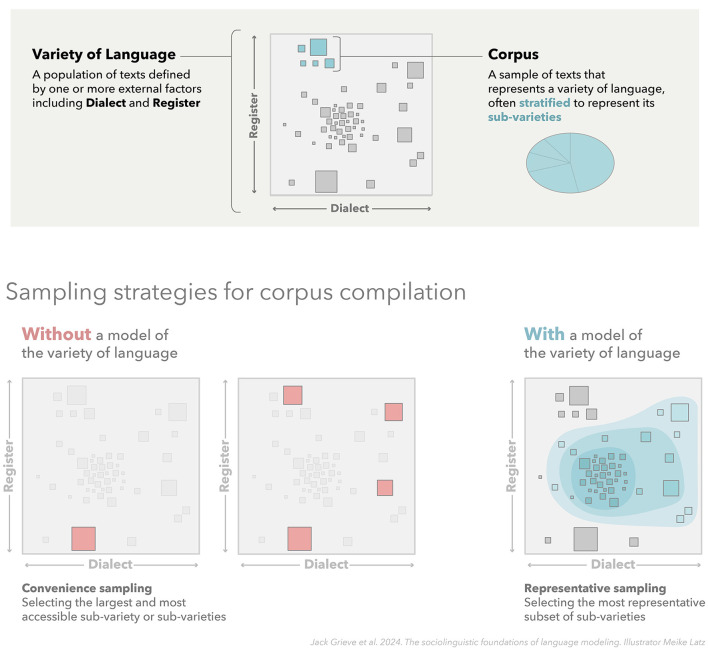
Representative corpus design. This figure presents a corpus as a representative sample of texts taken from a given variety of language (i.e., from a larger population of texts delimited by relevant extra-linguistic factors). This figure also illustrates how compiling a corpus that accurately represents a target variety requires access to an underlying model of that variety of language, including its internal sub-varieties, so that the corpus can be stratified so as to capture internal variation in that variety. Naive corpus compilation strategies that rely on convenience sampling will generally lead to less representative samples.

This conception of a variety of language is especially common in corpus linguistics, where a corpus is often seen as representing a variety of language: a corpus consists of a sample of texts drawn from the larger population of texts targeted for analysis (Biber, 1993; McEnery and Wilson, 2001; McEnery et al., 2006; Scholz et al., 2024). The goal of analyzing the structure of language observed in a corpus is therefore to draw generalizations about the variety of language (i.e., the larger population of texts) represented by that corpus. Furthermore, the quality of a corpus, and by extension the generalizability of any analyses based on that corpus, depends directly on the representativeness of this sample, including the accurate identification of its primary constituent sub-varieties. This relationship between sociolinguistic variation and corpus design is illustrated in Figure 2, which shows how a corpus can be seen as a representative sample of texts taken from a larger population of texts delimited by relevant extra-linguistic factors. This figure also shows how compiling a representative corpus in a principled manner generally requires access to an underlying model of that variety of language, including its internal sub-varieties, so that the corpus can be stratified so as to accurately represent internal variation in that variety. Without such a model, a corpus may misrepresent the patterns of linguistic variation that characterize a variety of language.

Finally, if a variety of language is defined as a population of texts delimited by some set of external criteria, the general expectation is that this population of texts will differ from populations of texts delimited by other external criteria in terms of its linguistic structure, including its grammar, phonology, lexis, and discourse (Crystal and Davy, 1969; Jackson, 2007). For example, among other features, a regional dialect may be characterized by the specific pronunciation of certain vowels (Labov et al., 2006), whereas a conversational register might be characterized by its rate of use of certain pronouns (Biber and Conrad, 2019). Crucially, we can expect that any social group or any social context that is recognized within society will generally become associated with distinct patterns of linguistic variation over time. At the most basic level, this is because certain words associated with concepts of particular importance to that group or context will be favored or will develop over time, although differences can generally be expected to emerge across all levels of linguistic analysis, depending on the communicative constraints and affordances associated with the extra-linguistic factors that define that variety (see Grieve, 2023). Although the number of possible varieties is therefore innumerable, a general goal of linguistic analysis is to identify varieties that are maximally distinctive, for example, mapping the dialect regions of a country (Wieling and Nerbonne, 2015; Grieve, 2016), defining the sub-types of a given register (Biber, 1989; Grieve et al., 2010), or identifying the most distinct periods of a language (Gries and Hilpert, 2008; Degaetano-Ortlieb and Teich, 2018).

To summarize the discussion presented in this section, we offer the following definition of a variety of language (see Figure 1):

A **variety of language** is a population of texts defined by one or more external factors, especially related to the social background of the people who produce these texts, the social context in which these texts are produced, and the period of time over which these texts are produced.

Furthermore, we define a **corpus** as a sample of texts drawn from a specific variety of language, i.e., from a larger population of texts (see Figure 2). In this sense, we say that a corpus *represents* a given variety of language. It is also important to stress, especially in the context of language modeling, that any corpus—any sample of texts—inherently represents some variety of language, namely, the smallest common variety that encompasses that sample of texts. However, the representativeness of any corpus depends directly on the quality and the size of the sample, as well as the accurate identification of the variety and its sub-varieties from which texts are sampled. For example, a sample consisting of a few conversational transcripts and emails collected in Great Britain could be taken as representing British English, just not very well.

Our primary contention in this paper is that, in general, language models, which are trained on large corpora of natural language, are inherently modeling varieties of language. In other words, we conceive of language models as models of *language use*—models of how language is used to create texts in the variety of language that the corpus used to train the model represents. Furthermore, like all linguistic models that are based on corpora of natural language, we believe that the validity and value of a language model depends on the degree to which the training corpus accurately represents the variety that is effectively being modeled, which we refer to as the **target variety**—even if that variety of language is unknown or under-specified.

Consequently, our claim is that understanding how to define and represent varieties of language is of direct relevance to language modeling: we believe that many problems that arise in language modeling result from a mismatch between the variety of language that language models are effectively intended to represent and the variety of language that is actually represented by the training corpora. We believe that this perspective is not only novel but fundamental to understanding the nature of language modeling and how to maximize the societal value of LLMs. To support and exemplify this claim, in the remainder of this paper, we therefore consider specific implications of this sociolinguistic conception of language modeling for a range of different challenges currently being faced in language modeling primarily through a critical review of the NLP literature from the sociolinguistic perspective introduced in this section.

## 3 Challenges

### 3.1 Social bias

**Figure 3:**
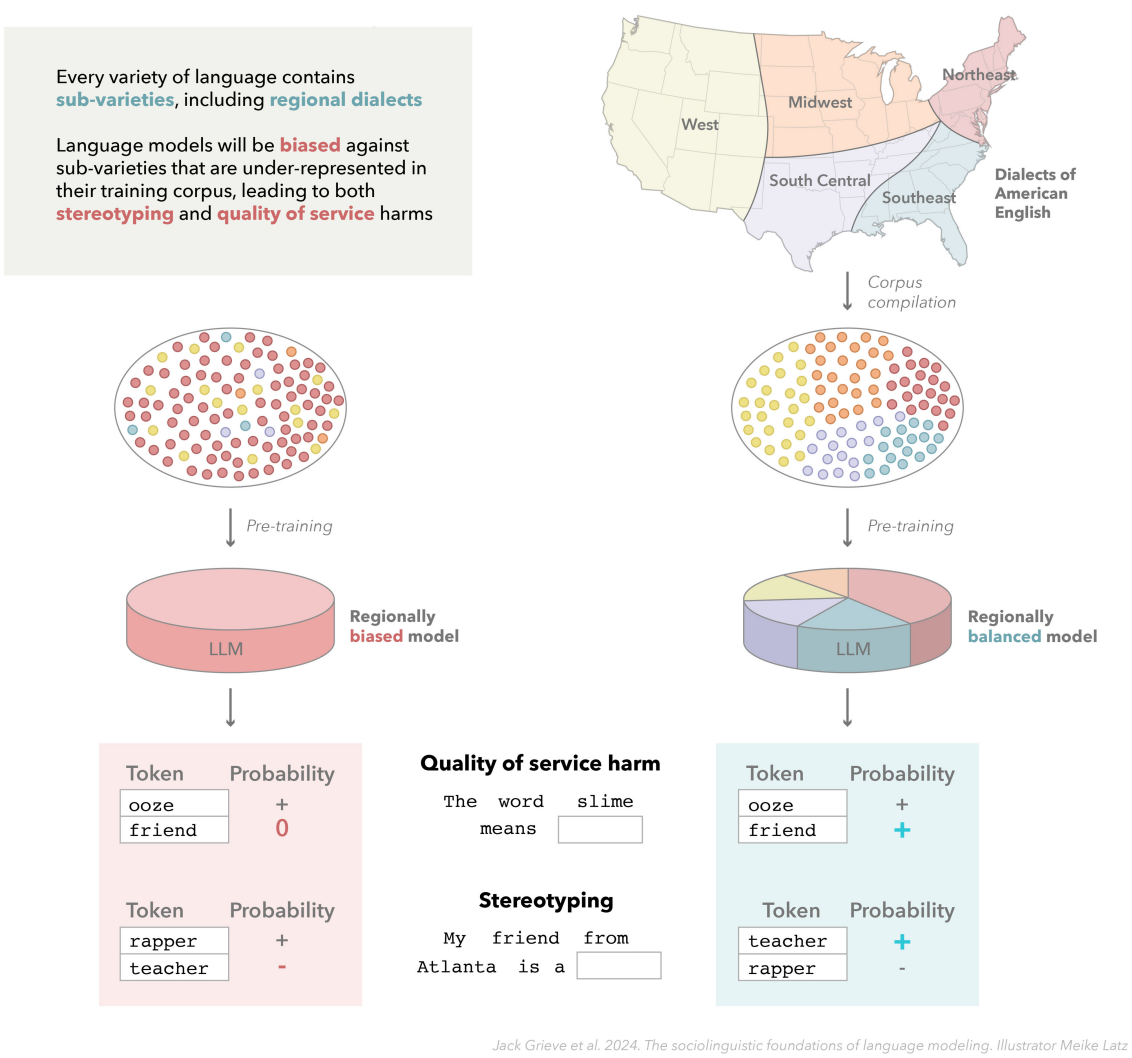
Sociolinguistic bias in language models. This figure illustrates how training language models on corpora that accurately represent the target variety of language including its internal structure, especially its constituent dialects, can potentially help address social bias, including both quality-of-service harms and stereotyping. This is exemplified by comparing two hypothetical models of American English, which are trained on corpora that inaccurately and accurately represent regional dialect variation (based on Grieve, 2016) in this variety of language.

NLP systems generally suffer from *social bias*: their real-world application leads to outcomes that unfairly disadvantage or harm specific social groups (Shah et al., 2020; Blodgett et al., 2020; Dev et al., 2022; Navigli et al., 2023; Luo et al., 2024). Social bias can be introduced at various points during the development and deployment of NLP systems (Hovy and Prabhumoye, 2021), but given the unsupervised nature of language modeling, training corpora are a key source of social bias in LLMs (Bender et al., 2021; Ferrara, 2023). While bias in NLP systems can harm people in various ways (Blodgett et al., 2020), in this section, we primarily focus on two common harmful outcomes of social bias. These two types of harms are most commonly discussed in terms of *quality-of-service harms* and *stereotyping harms* (e.g., Crawford, 2017; Blodgett, 2021; Dev et al., 2022; Weerts, 2021; Leidinger and Rogers, 2024; Chehbouni et al., 2024; Hofmann et al., 2024), although many different systems have been proposed for classifying biases and harms in NLP, which define these terms in somewhat different ways, along with many additional and often overlapping categories (Blodgett et al., 2020). Both of these types of harms are especially relevant to LLMs, and crucially we believe both can be better understood and addressed in language modeling by adopting a sociolinguistic perspective (see Figure 3).

First, social bias can be characterized by poor system performance for certain social groups that are interacting with language models and applications based on language models: token prediction will be more or less accurate depending on the social origins of the language inputted into the system. For example, ChatGPT might have difficulty correctly understanding prompts written by people from certain social groups due to their use of non-standard or socially restricted language patterns. This type of bias leads to what is known as **quality-of-service harms**, where the performance of these systems varies depending on the social background of the user (Crawford, 2017; Dev et al., 2022; Chehbouni et al., 2024). These types of quality-of-service harms can often be the product of **selection bias**, as they result from how training data is *selected* from across the society whose language is being modeled (Shah et al., 2020): in general, if language data from certain social groups is under-represented in the training data for a language model, we should expect that applications of that model will process language structures produced by these groups less accurately and consequently exhibit poorer performance for these groups (Blodgett et al., 2020; Lahoti et al., 2023).

Notably, quality-of-service harms, especially those resulting from selection bias, have been one of the central concerns in computational sociolinguistics (Nguyen et al., 2016; Eisenstein, 2017; Grieve et al., 2023). Researchers in this emerging field have stressed for the past decade that the performance of NLP systems generally varies for people from different social groups and have called for engagement with description and theory from sociolinguistics to help address this basic form of social bias (e.g., Hovy and Søgaard, 2015; Jórgensen et al., 2015; Blodgett and O'Connor, 2017; Jurgens et al., 2017; Schramowski et al., 2022; Hofmann et al., 2024).

Second, social bias can be characterized by systems that produce outputs that directly harm or discriminate against certain social groups even when they are not directly engaging with these systems themselves. For example, when prompted, ChatGPT might be more likely to produce negative portrayals of certain ethnicities and genders, no matter who is doing the prompting (Bommasani et al., 2021; Lahoti et al., 2023). Most notably, this type of bias can lead to what is known as **stereotyping harms** (Crawford, 2017; Leidinger and Rogers, 2024; Hofmann et al., 2024), as well as related harms like *disparagement* and *dehumanization* (Dev et al., 2022), where negative viewpoints about specific social groups are propagated, as has been widely discussed in regards to LLMs (Bender et al., 2021). Once again, it is clear that this issue can be traced back, at least in part, to the data the language model was trained on. If the training corpus contains relatively frequent expression of harmful or inaccurate ideas about certain social groups—as we can safely assume any large, unconstrained sample of internet writings will—language models will inevitably reproduce those biases (Bender et al., 2021; Ferrara, 2023; Hofmann et al., 2024). As Bender et al. (2021, 613) state, “large, uncurated, Internet-based datasets encode the dominant/hegemonic view, which further harms people at the margins.” These types of harms are generally the product of **semantic bias**, as they result from the meaning relationships between words inferred by the language model based on patterns of co-occurrence observed in the training corpus (Shah et al., 2020).

From a sociolinguistic perspective, we believe social bias in language models can be addressed at a basic level by pretraining on corpora that more accurately represent the target variety. Imbalance in pretraining data is a recognized as a general source of social bias in language modeling (Yogarajan et al., 2023; Kocijan, 2021; Hofmann et al., 2024). Although social bias can be partially detected or resolved by manipulating the embedding space (Caliskan et al., 2017), the probability table (Salazar et al., 2019), or the output of the text generation process (Bordia and Bowman, 2019), these approaches have numerous limitations. For example, models that are de-toxified following pretraining will tend to generate less content about the social group that had been the target of toxic discourse, inadvertently leading to the erasure of that social group (Xu et al., 2021). More generally, these types of interventions all fall outside the basic language modeling task, focusing on suppressing bias-related parameters (Liu et al., 2024), rather than pretraining better underlying language models. To address bias in language models at a fundamental level requires intervention at the pretraining stage (Yogarajan et al., 2023; Hofmann et al., 2024). Our claim is that this type of intervention can be pursed in a principled manner by pretraining on corpora that accurately represent the target variety of language, as identified through sociolinguistic analysis.

Furthermore, we believe that it is especially important that the training corpus represents the *internal structure* of the target variety, in the sense that the sub-varieties of that variety of language, including most importantly the major dialects of that variety of language, are adequately represented in the training corpus, reflecting both the size and distinctiveness of those dialects. This challenge is illustrated in Figure 3, which shows how a language model for American English could be biased toward one regional dialect or biased against another in various ways. For example, a corpus intended to represent American English, but which is primarily composed of texts collected from a specific dialect of American English (e.g., texts written by highly educated, middle-class, white Americans from major coastal cities), cannot adequately represent the full diversity of American English. Any language model trained on such a corpus should therefore be expected to be biased against social groups that are underrepresented in the training data, such as African American English from the Southern US, compared to a language model trained on a corpus that more accurately represents variation in American English.

The link between corpus design and quality-of-service harms in LLMs is especially clear: because language varies in systematic ways, to ensure a language model can accurately process language *from* a wide range of social groups, it should be trained on corpora that represent the language used *by* a wide range of social groups, i.e., their dialects, as illustrated in Figure 3. For example, consider lexical variation in British and American English: if a model were only trained on American English, it would be much more likely to misinterpret the meaning of words that tend to have different meanings in British English, like *boot* (for *trunk*) or *underground* (for *subway*). Consequently, the quality of service provided by applications based on that model for speakers of British English would be degraded.

Stereotyping and related forms of discrimination generated by LLMs have also often been traced back to issues with data collection and curation (Bender et al., 2021). A sociolinguistic perspective potentially provides a principled solution to this problem: in general, stereotyping harms could be addressed by training on data that better represents the language produced by a wider range of social groups. One reason that certain social groups are negatively portrayed by LLMs is that they are not allowed to portray themselves, in their own words, in the data used for training. By training on corpora that equitably and deliberately represent the internal varietal structure of the target variety, especially the range of dialects of which it is composed, we believe that stereotyping and other forms of semantic bias can be mitigated (see Figure 3). In other words, modeling data from a wider range of dialects—and, by extension, from a wider range of social groups—would help ensure that a wider range of viewpoints would be represented by a language model. Stratified corpora that accurately represent the sociolinguistic structure of the target variety (i.e., its constituent sub-varieties) could also potentially be used to evaluate and probe a model, allowing for social bias to be identified and interpreted directly.

The sociolinguistic approach to language modeling advocated for in this paper therefore provides a simple yet theoretically grounded basis for understanding the general source of social bias in language modeling, including for addressing both quality-of-service and stereotyping harms, as well as other related types of harms. In addition, a sociolinguistic approach offers a clear pathway for both interpreting and addressing these different forms of social bias during pretraining through careful corpus compilation informed by scientific understanding of the nature of linguistic variation within that specific target variety, based either on existing or new sociolinguistic research. Crucially, however, such sociolinguistic interventions need not necessarily occur during the *initial pretraining* of the base model, but can be pursued through the *further pretraining* of base models, as we discuss in the next section.

### 3.2 Domain adaptation

Despite their remarkable fluency and general applicability, LLMs generally benefit from some form of **domain adaptation** before deployment (Radford et al., 2019; Gururangan et al., 2020). In NLP, domain adaptation is the task of improving the performance of a system that was developed using language data collected in one domain for a different and often more specific domain where the system is to be applied—the real—world context where the system is used, such as texts about a particular topic or from a particular genre (Daumé, 2007). Although there are many approaches for adapting language models, including for different downstream tasks—including reinforcement learning from human feedback (Ouyang et al., 2022), low-rank adaptation (Hu et al., 2021), and low-tensor rank weight adaptation (Bershatsky et al., 2024)—we focus on the process of fine-tuning a base model by extending unsupervised language modeling on a corpus of texts sampled from a specific target domain (Gururangan et al., 2020; Hu et al., 2021; Hou et al., 2022).

This approach is often referred to as **further pretraining** because it involves extending the basic form of unsupervised language modeling used to train the base model to new data from the more specific target domain (Gururangan et al., 2020). The goal is simply to improve the accuracy of token prediction in the target domain, while preserving the underlying fluency of the base model. For example, a base model trained on huge amounts of unrestricted online language data could be adapted to the specific domain of customer service: based on a corpus of customer service transcripts, the parameters of the base model would be adjusted to improve the ability of the model to predict word tokens in texts from that domain given the topics of discussion and the specific types of interactions that characterize that domain (Chen et al., 2024). In practice, further pretraining has been proven to be an effective way of improving the performance of LLMs across a wide range of downstream tasks, including medical text processing (Lehman et al., 2023; Nazi and Peng, 2024), cross-lingual transfer (Aggarwal et al., 2024), and named-entity recognition in low-resources domains (Mahapatra et al., 2022).

**Figure 4:**
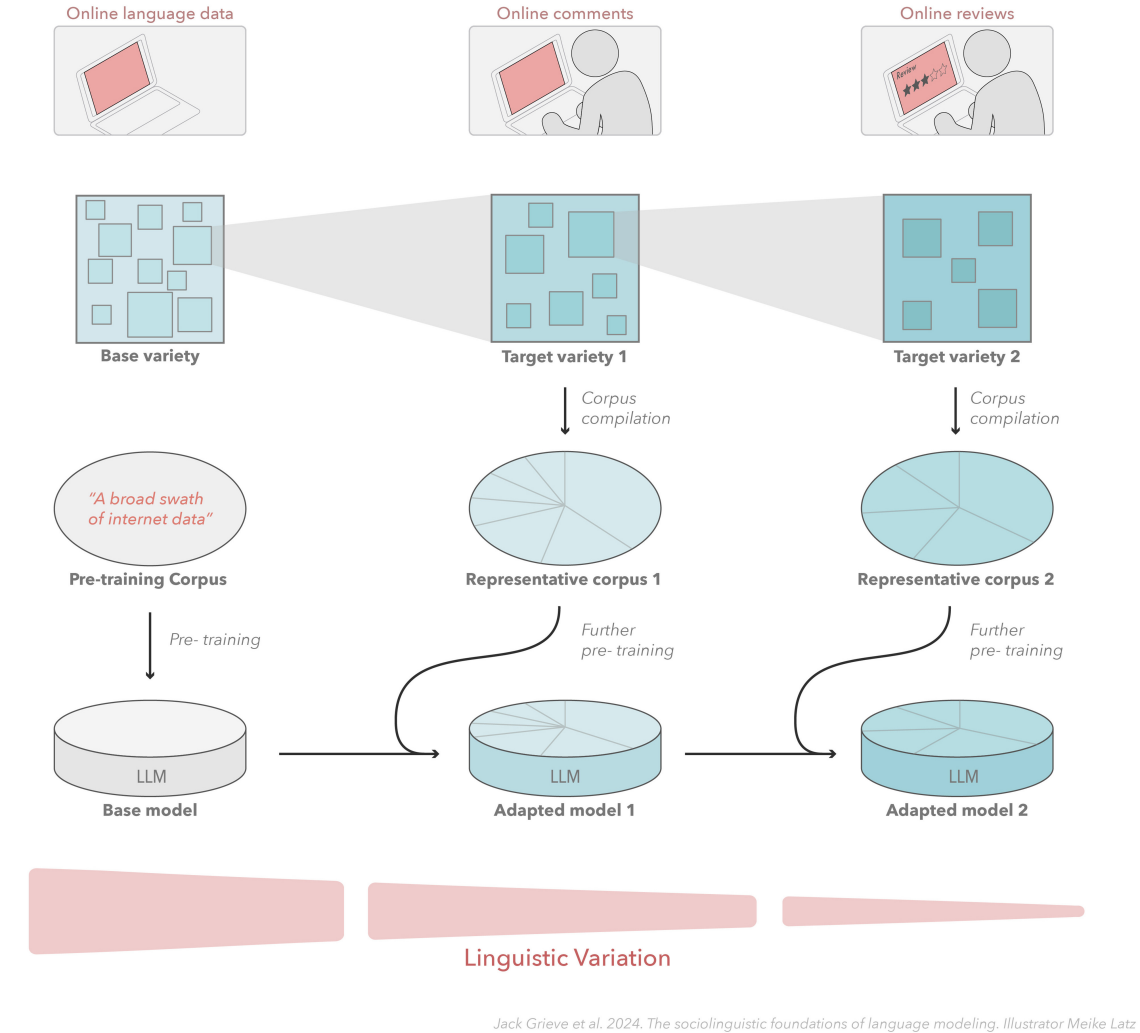
Sociolinguistic adaptation of language models. This figure illustrates how an understanding of the sociolinguistic structure of varieties of languages can inform the adaptation of language models. Language model adaptation can be seen as the process of fine-tuning a base model, potentially in an iterative manner, to predict word tokens in a more narrowly defined variety of language that is subsumed by the larger variety of language represented by the base model.

Although the importance of domain adaptation has long been appreciated in language modeling (Rudnicky, 1995; Chen et al., 2024), we believe that this process can be reframed directly and insightfully in sociolinguistic terms, where domain is understood as a variety of language. If the goal of the base model is seen as accurately predicting word tokens in a broadly defined variety of language, like the English language, then the goal of domain adaptation can be seen as the process of fine-tuning the base model to allow it to predict word tokens more accurately in a more narrowly defined variety of that language—the sub-variety associated with the target domain. Crucially, the adapted model should be expected to be more accurate because more narrowly defined varieties of language *must* be characterized by less variation than any larger variety that encompasses it. This process can also potentially be carried out in an iterative manner, where a base model is repeatedly adapted on corpora representing more narrowly defined varieties of language, as shown in Figure 4, which illustrates a sociolinguistically informed approach to domain adaption, where a model is iteratively fine-tuned on corpora representing increasingly narrowly defined varieties of computer-mediated communication.

A sociolinguistic perspective on domain adaptation therefore sees the target domain as a variety of language. This means that the process of domain adaptation can be informed by linguistic analysis that rigorously identifies maximally distinctive varieties of language. This can include both existing research in sociolinguistics, dialectology, and related fields, as well as new research conducted directly to support model training for specified domains. For example, if a base model is adapted for a specific region of the US, empirical research in American dialect geography (e.g., Grieve, 2016) should be consulted to precisely define the sub-region that is being targeted for adaptation (see Figure 3). Similarly, if a base model is adapted for a specific type of blog writing, empirical research on register variation in blogs (e.g., Grieve et al., 2010) should be consulted to precisely define the sub-type of blog writing that is being targeted for adaptation. Notably, recent research in NLP has begun to offer empirical evidence for the value of this approach in downstream tasks. For example, in hate speech detection, adapting the underlying language models to what are effectively target dialects (Pérez et al., 2024) and registers (Nirmal, 2024) has been found to lead to improvements in the overall performance of these systems.

Crucially, sociolinguistics does not only provide a basis for identifying valid targets for domain adaptation but for mapping and modeling the internal structure of these target varieties. This is especially important because target varieties for domain adaptation are often well-defined by default. For example, if a fine-tuning corpus is collected by sampling data from a particular social media platform, a relatively homogeneous variety of language will have naturally been targeted; however, a random sample of texts from that variety, drawn without taking into account its internal structure, might severely under-represent sub-varieties of interest. For example, a social media corpus may be dominated by certain sub-registers (e.g., abusive or promotional posts) that are not the intended target of adaptation, while the sub-registers that are the intended target of adaptation (e.g., interactive or informational posts) may be limited. Similarly, people from certain social groups may be underrepresented in specific domains, resulting in social bias being inadvertently exacerbated by naive domain adaptation. In many cases, the target variety cannot even be accurately defined until the overall structure of the larger variety in which it is subsumed is understood through careful sociolinguistic analysis.

A sociolinguistic perspective also highlights a more general problem with domain adaptation: the success of this process depends on the relationship between the larger variety represented by the base model and the smaller target variety toward which the base model is being adapted. Ideally the variety of language represented by the base model would completely subsume the target variety: the target variety would be a sub-variety of the base variety, regardless of whether it was represented directly in the base training data. However, the target variety may not be adequately represented in the data sampled for training the base model. For example, the target variety could be associated with a social group or a social context that is severely underrepresented in the base training corpus. In such situations, fine-tuning regimes informed by sociolinguistic theory and description would likely be beneficial by providing a basis for identifying these varieties and sampling language directly from these contexts and communities.

Understanding the sociolinguistic structure of the larger variety of language could also allow models to be adapted to represent target varieties with missing data. If empirical research in linguistics has found that a target dialect or register for which data is lacking falls between multiple dialects or registers for which data is available, a model could be adapted for the target variety by training on a combination of the available corpora. Overlap between varieties could also be exploited in a similar way: for example, if data is lacking for a target variety defined in terms of a specific register and a specific dialect, a model could be adapted for the target variety by fine-tuning on a combination of corpora that represent that specific register and that specific dialect. These types of techniques could even be used to create a model of a variety of language that does not yet exist—engineered by training on corpora representing different registers and dialects.

Finally, it is important to stress that our proposal is not meant to be a simple solution to the problem of domain adaptation that can be applied mechanically or without sociolinguistic expertise. Given the complexity of language variation and change, we do not believe such an approach is possible. A sociolinguistic approach to domain adaptation must draw upon detailed empirical research on that specific variety of language and its constituent sub-varieties to direct the compilation of representative training corpora. If this empirical research has already been conducted by sociolinguists, it can be consulted directly, but if no such research exists, new sociolinguistic research would need to be conducted. Although this research would be grounded in general methods for sociolinguistic analysis, the results would necessarily be specific to that variety of language.

### 3.3 Alignment

The challenges of social bias and domain adaptation can be seen as forms of the more general *alignment problem*—how to ensure that the behavior of AI systems aligns with the values and expectations of society (Gabriel, 2020; Hendrycks et al., 2020; Christian, 2021; Ngo et al., 2022; Dung, 2023). Misalignment arises not simply when AI systems fail to achieve their intended goals, but when they pursue these goals, even successfully, in ways that have negative or unforeseen consequences or that are not in accordance with societal values, for example, in ways society finds to be inappropriate, unethical, immoral, or dishonest. **Alignment** is therefore the general process of guiding AI systems to behave in ways that are consistent with the broader expectations of society, while discouraging them from behaving in ways that are inconsistent with these expectations, especially to avoid unintended risks and harms (Russell and Norvig, 2016). Crucially, the challenge is not only *how* to guide AI systems but *where* to guide them (Gabriel, 2020).

Although alignment is a long-standing concern in AI (Wiener, 1960), attention has grown in recent years due to the growing complexity and ubiquity of real-world AI systems, especially systems based on language models (Shen et al., 2023; Liu et al., 2022, 2023; Wang et al., 2023; Wolf et al., 2023), which potentially allow for misalignment to emerge on many different levels (Gabriel, 2020; Dung, 2023). For example, consider a generative language model that automatically produces reviews of scientific literature on a specified topic. An obviously misaligned system might produce reviews that are clearly wrong—incoherent or incorrect—while a less obviously misaligned system might produce fluent reviews, completing the task successfully in a superficial way, but getting facts wrong, for example, referencing publications that do not exist. This type of a *hallucination*—the presentation of false information as if it is true—is a common form of misalignment in LLMs (Evans et al., 2021; Tonmoy et al., 2024). A more insidiously misaligned system, however, might produce perfectly accurate and fluent syntheses that cite relevant literature, but exhibit other problematic behaviors, such as limiting references to certain ideas or researchers in certain fields, thereby effectively suppressing certain viewpoints (Bender et al., 2021).

A basic approach for aligning language models involves pretraining or further pretraining on corpora that are considered to be more aligned with the values and expectations of society (Solaiman and Dennison, 2021). How such corpora can best be compiled, however, is far from clear. As we have argued throughout this paper, sociolinguistic theory provides a basis for compiling better training corpora. In the general case of alignment, we believe language models can be aligned with the values and expectations of society, crucially without pre-specifying what exactly these values and expectations are, by training on corpora that accurately represent the range of varieties found in that society. As discussed in terms of social bias, language models can be trained to better align with the general values of a society, as opposed to the values of some particular social group within that society, by balancing training data originating from different dialects. Similarly, as discussed in terms of domain adaptation, language models can be trained to better align with expectations that they will perform adequately across the range of communicative contexts found in that society by balancing training data originating from different register. This is because the values and expectations of a society are instantiated in their patterns of language use.

In addition to addressing specific alignment issues related to social bias and domain adaptation, we believe a sociolinguistic approach can potentially help us train models that are less susceptible to unethical and dishonest behaviors in general (Huang C. et al., 2024). This is because respecting sociolinguistic diversity entails training models on data that represents a greater diversity of viewpoints, experiences, and opinions. As LLMs are models of varieties of language, they will be better models, more aligned with the needs, expectations, and values of society, when they account for the full range of sub-varieties, and hence the full range of perspectives, found within that society. In general, we therefore believe that a major source of LLM misalignment comes from what we call **varietal misalignment** and that LLM misalignment can therefore be addressed, at least in part, by compiling training corpora to accurately represent the varietal structure of the target variety, as identified through sociolinguistic analysis.

Finally, it is important to acknowledge that while a sociolinguistic perspective can provide a basis for aligning language models for the society that it is intended to serve, this approach does not ensure that the resultant language models will be aligned with the ethical and moral *aspirations* of that society. For example, a generative language model trained on a socially balanced corpus of the English language will still potentially produce texts that express racist viewpoints because a portion of English texts expresses racist viewpoints. There might be greater equity in the types of stereotypes it spreads, but such behavior can still be seen as a form of misalignment. A sociolinguistic perspective, however, also provides a possible solution to this problem—by deliberately weighting the varieties of language represented in the training corpus. For example, if a particular social group has been broadly disadvantaged or has a worldview that society wishes to encourage, the portion of the corpus representing the relevant varieties of language can be more heavily weighted during pretraining or further pretraining. In this way, a sociolinguistic perspective can provide a theoretical basis not only for *balancing* but for *controlling* the alignment of language models.

### 3.4 Language change

Thus far, our discussion has focused on how a series of challenges in language modeling related to bias, adaptation, and alignment more generally can be addressed, in principle, by building training corpora that better represent the dialects and registers of the target variety. Another form of this basic problem involves ensuring that language models and applications based on language models are responsive to language change and cultural change more generally (Bender et al., 2021; Bommasani et al., 2021). All varieties of language change over time, often in ways that are difficult, if not impossible, to predict (Lass, 1997). If language models are to maintain their fluency and not become obsolete, they must therefore be continuously updated using training corpora that consist of examples of contemporary language use. In principle, this problem can be resolved by compiling new corpora over time that *consistently* represent the target variety and its evolving internal varietal structure. The challenge is therefore to understand how the sociolinguistic landscape of registers and dialects within that variety of language has changed over time, which can only be accomplished accurately through detailed and ongoing sociolinguistic analysis.

A related issue that has caused growing concern in language modeling is that over time more and more real-world language will presumably be produced with the assistance of LLMs, which will make it increasingly difficult to compile contemporary corpora of *real* human language for training new models or updating existing ones (Shumailov et al., 2023). Proposed solutions to these problems of *data contamination* (Balloccu et al., 2024) and *task contamination* (Li and Flanigan, 2024) generally involve finding ways to exclude machine-generated language from future training data, including through watermarking systems (Kirchenbauer et al., 2023; Dathathri et al., 2024). These types of solutions, however, would seem easy to confound, if only because they do not generally allow texts written collaboratively by human and machine to be identified, which is likely to become increasingly common and diversified in everyday life.

Despite real concerns about LLM detection in certain contexts (Bommasani et al., 2021; Bian et al., 2023), the rising use of LLMs to generate language is not difficult to reconcile with sociolinguistic theory and practice. Over time, AI systems based on language models will undoubtedly start to change how we use language. Texts generated with the help of language models will increasingly enter into the real world. At this point, from an externalist perspective (Scholz et al., 2024), these texts will be part of language—produced, transmitted, and understood by humans as language, often indistinguishable from human-generated language in the regular flow of real-world language use. Ultimately, the distinction between human- and machine-generated language can therefore be seen as simply another aspect of register that defines variation within varieties of language, just like all communicative technologies that have come before, including the invention of writing and digital communication.

Taking a sociolinguistic perspective, it is also important to acknowledge that the rise of language models is creating *new* varieties of language, including those characterized by the linguistic interaction between humans and machines, such as dialogues with ChatGPT (Mavrodieva, 2023). These new varieties, which will only continue to diversify over time, will also need to be accounted for, like all varieties of language, both by theories of sociolinguistic variation and by the evolving language models designed to represent contemporary language use. If language models are to be kept up-to-date, machine-generated language cannot be excluded, as its production will become a significant driver of language change.

### 3.5 Scale

In addition to more specific insights into the development and deployment of language models, we believe a sociolinguistic perspective can also help to explain the remarkable success of LLMs, which has been attributed both to the development of new deep learning architectures and the use of extremely large corpora of natural language for pretraining (Kaplan et al., 2020; Bender et al., 2021; Bommasani et al., 2021). Although there is a clear relationship between the scale of the training data and the success of these systems (Sardana and Frankle, 2023; Hoffmann et al., 2022; Bahri et al., 2024), it is unclear *why* increasing the amount of training data results in such great increases in performance. Is there a limit to how much performance can be gained simply by increasing the scale of the training data? How can more powerful models be developed with less data? These are fundamental questions for LLM development (Bommasani et al., 2021), especially because of the significant costs and environmental impacts associated with increases in scale (Bender et al., 2021). We believe these are questions that can be uniquely informed by a sociolinguistic perspective.

The obvious reason why increasing the amount of training data provided to a language model improves its performance is that this provides the model access to a wider range of language patterns (Shumailov et al., 2023). This is presumably why LLMs benefit from being pretrained on such large corpora of natural language: the same levels of performance could not be achieved by pretraining twice as long on half the data. Scale is therefore not sufficient on its own. What matters is not simply the *scale* of the training data but the *diversity* of the training data. Although the importance of the diversity of training data has often been stressed in critical discussions of LLMs (Brown et al., 2020; Bender et al., 2021), the sociolinguistic perspective advocated in this paper provides a theoretical basis for understanding this relationship with greater precision: diversity in the training corpus, in terms of both its linguistic structure and its semantic content, can be seen as directly reflecting the diversity of the varieties of language represented by that corpus. To maximize the performance of language models and the efficiency with which these improvements can be obtained, we therefore believe it is more important to prioritize the amount of varietal diversity in the training data over scale. This can be achieved by carefully representing a wider range of varieties in the training data, including both dialects and registers, grounded on empirical sociolinguistic analysis of the target variety and its internal patterns of linguistic variation.

Notably, empirical evidence for prioritizing diversity in training data in language modeling is building. In addition to research on debiasing (Hofmann et al., 2024) and domain adaptation (Gururangan et al., 2020) that has stressed the importance of further pretraining on diverse data, the superior performance of GPT-3 over GPT-J—both of which share the same base model architecture—provides an especially clear evidence of the importance of diversity over scale (Wang and Komatsuzaki, 2021; Brown et al., 2020). GPT-3 is generally considered to have benefited from OpenAI's carefully curated, even if largely undocumented, training dataset, whereas GPT-J was pretrained on an open data set called *the Pile* (Gao et al., 2020), which is presumably far less carefully curated. Another source of evidence for the importance of diversity in training data is the rapid degradation of model performance and breaks in information integrity that have been found to occur when LLMs are trained on data generated by other LLMs, which is inherently far less diverse than language produced by humans (Shumailov et al., 2023), as has been demonstrated repeatedly in recent research on LLM detection (Bevendorff et al., 2024; Huang and Grieve, 2024).

A sociolinguistic perspective provides a basis for assessing the diversity of training data and the effect of varying the diversity of training data along multiple dimensions on the performance of the resultant models in a meaningful way. For example, there is considerable research on quantifying the overall degree of linguistic diversity and complexity in corpora in both dialectology (Wieling and Nerbonne, 2015; Röthlisberger and Szmrecsanyi, 2020) and register analysis (Ehret, 2021; Biber et al., 2021).

This sociolinguistic perspective also provides an answer to questions about the limits of increasing the scale of training data (Bommasani et al., 2021). At what point should increasing the size of the training corpus no longer lead to substantial improvements in model performance? Our hypothesis is that increasing the scale of training data will continue to increase the performance of language models so long as it also results in an increase in the sociolinguistic diversity in the training corpus. Crucially, this implies that attempts to empirically assess the limits of scale simply by comparing model performance as the amount of training data increases will not be accurate, unless the sociolinguistic diversity of the corpus is also controlled for and measured alongside corpus size (Hoffmann et al., 2022).

This insight is directly relevant to defining *scaling laws* (Bahri et al., 2024) for language models (Bommasani et al., 2021), which are attempts to specify how much data is needed to train a language model with a given number of parameters. This issue has most famously been discussed in terms of what is known as the *Chinchilla Law*, which states that, for each parameter in an LLM, 20 tokens of training data is optimal (Hoffmann et al., 2022). By this standard, GPT-3, for example, is much too large given the amount of training data. From a sociolinguistic perspective, however, any such calculations seem overly simplistic, as they ignore the diversity of the training data. This issue has not been entirely missed in language modeling. For example, the Chinchilla Law assumes the training data is of "high quality", although exactly what this means and how this can be assessed is a largely unexplored topic (Sardana and Frankle, 2023). Measuring the overall degree of sociolinguistic diversity in training data can provide a basis for making these types of assessments.

Finally, a sociolinguistic perspective also offers clear direction for training models using limited amounts of data. This is especially important issue when the goal is to build language models for under-resourced varieties of language, where obtaining sufficiently large corpora for training models is a major challenge (Bender et al., 2021; Ramesh et al., 2023). Specifically, if the value of training data is largely determined by the diversity of training data, great care should be taken to maximize the amount of sociolinguistic diversity, both in terms of dialect and register variation, in the data used to train language models for under-resourced varieties.

## 4 Conclusion

In this paper, we have proposed that, in general, language models inherently represent varieties of language. Our claim is that whenever tokens are predicted based on the observation of linguistic patterns in corpora of natural language, the resultant language model is necessarily a model of the variety of language represented by that corpus. By extension, we have argued that the performance, utility, and ethical application of language models, as well as any NLP systems in to which the are embedded, depends on how well the corpora on which they are trained represent the varieties being modeled, including their internal varietal structure. In other words, we believe that the performance and societal value of language models is determined not only by the amount of language data used for training but by the sociolinguistic diversity and representativeness of these corpora. Crucially, the arguments we have presented in this paper are intended to be relevant to any form of language modeling—not only current transformer-based models, but simpler traditional models, as well as future approaches to language modeling that have not yet been developed.

For these reasons, we believe that drawing on insights from sociolinguistics to direct the design, compilation, and curation of training corpora will be critical to the future of language modeling, with widespread implications for their development and deployment. Specifically, we have identified and discussed several challenges in language modeling—social bias, domain adaptation, alignment, language change, and scale—that we believe a sociolinguistic perspective could help address in a principled and unified manner. Although our goal in this paper has been to introduce this new perspective on language modeling through a theoretical discussion grounded in existing research in sociolinguistics and NLP, we hope our proposal will act as a foundation and inspiration for future empirical research in this area, not only in NLP but in linguistics (Huang W. et al., 2024; Huang and Grieve, 2024).

It is also important to acknowledge that there already has been considerable discussion of these types of challenges in language modeling and NLP more generally, with proposals to address these issues often emphasizing the need for more careful curation of training data (Bender et al., 2021; Hovy and Prabhumoye, 2021) and for incorporating social and even sociolinguistic insight into these models (Hovy, 2018; Hovy and Yang, 2021; Nguyen et al., 2021; Yang et al., 2024), especially within the emerging field of computational sociolinguistics (Nguyen et al., 2016; Grieve et al., 2023). For example, to address risks related to social bias in LLMs, Bender et al. (2021, p. 610) recommend that resources must be invested for “curating and carefully documenting datasets rather than ingesting everything on the web,” while Yang et al. (2024, p. 1) argue that issues with LLM performance are related to “a lack of awareness of the factors, context, and implications of the social environment in which NLP operates, which we call *social awareness*”.

What we believe is lacking in these discussions, however, is the identification of a general linguistic framework for solving these types of problems within the basic paradigm of language modeling, especially one that is theoretically grounded in our scientific understanding of language variation and change. Although the lack of social diversity in training data has been repeatedly identified as a problem for LLMs, what exactly this means and how exactly this can be measured and addressed in a principled manner has not been articulated. Given this emerging discourse, the primary contribution of this paper is to propose a theoretical and empirical foundation for addressing a wide range of challenges in language modeling that is based directly on sociolinguistic theory, specifically the concept of a *variety of language*—a topic that, to the best of our knowledge, has been absent from discussions of language modeling up until now, even within computational sociolinguistics. This perspective is also notably quite different from discussions of language modeling in linguistics, which have focused on the status of LLMs as *models of language cognition* (Piantadosi, 2023; Dentella et al., 2023; Marcus et al., 2023; Tsvilodub et al., 2024). In this article, we have attempted to shift this discussion, focusing instead on understanding language models as *models of language use*, which we believe has far more direct and immediate consequences for the development and deployment of language models in the real world.

Our basic claim is therefore that language models can be improved in many ways by training on datasets that endeavor to accurately represent the varieties of language being modeled. We therefore believe that there is a clear and urgent need for engagement with sociolinguistic research in language model design and evaluation. At the most basic level, language models are models of how language is used for communication within society. Understanding the structure of society, and how this structure is reflected in patterns of language use, is therefore critical to maximizing the benefits of language models for the societies in which they are increasingly being embedded.

Finally, in this paper, we have focused exclusively on the basic task of language modeling (i.e., pretraining and fine tuning via further pretraining). Our goal has been to explain how and why a sociolinguistically informed approach to the curation of training data can improve the societal value of language models in general. Nevertheless, we believe sociolinguistic insight, and linguistic insight more generally, can inform the broader development and application of modern LLMs, including improving approaches to reinforcement learning (Ouyang et al., 2022), prompt engineering, and in-context learning (Chen et al., 2023), all of which are ultimately grounded in patterns of language use. Moving forward, we therefore believe that research on language use—not only in sociolinguistics, but in corpus linguistics, discourse analysis, pragmatics, cognitive linguistics, and other fields of linguistics that focus on understanding how language is used for communication in the real world—will increasingly become central to advancing the field of language modeling, as well as NLP and AI more generally.
